# Mapping the Effectiveness of Programmed Intermittent Epidural Bolus Versus Continuous Epidural Infusion for Labor Analgesia: A Scoping Review

**DOI:** 10.7759/cureus.87143

**Published:** 2025-07-01

**Authors:** Gurulingappa I Herakal, Prashanth Kumar Challangod, Praveen Kumar Kandakurti, Prathima Shetty

**Affiliations:** 1 Department of Anesthesiology, Srinivas Institute of Allied Health Sciences, Srinivas University, Mangaluru, IND; 2 Department of Anesthesiology, Srinivas Institute of Medical Sciences and Research Center, Srinivas University, Mangaluru, IND; 3 College of Health Sciences, Gulf Medical University, Ajman, ARE; 4 Department of Anesthesiology and Operation Theater Technology, Nitte Institute of Allied Health Sciences, Nitte (Deemed to be University), Mangaluru, IND

**Keywords:** bupivacaine, epidural analgesia, fentanyl, labour and delivery, levobupivacaine, ropivacaine, sufentanil

## Abstract

Epidural analgesia is a cornerstone of labor pain management, utilizing continuous epidural infusion (CEI) or programmed intermittent epidural bolus (PIEB) to deliver local anesthetics combined with opioids. Hence, this scoping review maps the evidence on the comparative efficacy and outcomes of CEI versus PIEB in labor analgesia. A comprehensive search was conducted on PubMed, ScienceDirect, and Google Scholar databases for full-text English-language studies published between 2015 and March 2025. Inclusion criteria encompassed studies comparing CEI and PIEB in healthy term pregnant women, focusing on pain relief, motor block, maternal satisfaction, and obstetric outcomes. Overall, 11 studies were included, from which the results showed that PIEB consistently demonstrated superior pain relief, reduced motor block incidence, and higher maternal satisfaction. PIEB also required fewer anesthetics and fewer rescue boluses. Moreover, trends toward lower instrumental delivery rates and shorter second-stage labor duration were observed, although often statistically nonsignificant. Key research gaps include optimal dosing regimens, direct anesthetic comparisons, and long-term maternal and neonatal outcomes. In conclusion, PIEB appeared superior to CEI for labor analgesia, offering enhanced pain control, reduced motor block, and improved satisfaction. However, large, multicenter randomized controlled trials with standardized protocols and extended follow-up are needed to optimize PIEB and confirm its benefits across diverse populations.

## Introduction and background

During labor, epidural analgesia is a cornerstone for the management of pain while allowing women to actively participate and remain alert during the birthing process. The technique typically involves the administration of local anesthetics, such as levobupivacaine, bupivacaine, or ropivacaine, often combined with opioids like fentanyl or sufentanil, to achieve analgesia with minimal systemic effects [1]. Two primary methods of epidural drug delivery have emerged: continuous epidural infusion (CEI), which delivers a steady infusion of anesthetic to maintain consistent drug levels, and programmed intermittent epidural bolus (PIEB), which administers periodic boluses to potentially achieve better spread and efficacy within the epidural space [2]. The choice between CEI and PIEB can significantly influence pain relief, motor function, maternal satisfaction, and obstetric outcomes, yet the optimal approach remains a subject of ongoing debate [3].

Local anesthetics used in epidural analgesia differ in potency, duration, and motor block profiles, which may affect their performance in CEI versus PIEB regimens. For instance, PIEB is hypothesized to provide superior analgesia by promoting a more uniform distribution of anesthetic in the epidural space, potentially reducing breakthrough pain and motor block incidence compared to CEI [4]. However, variations in dosing regimens, anesthetic choice, and patient characteristics, such as parity or labor stage, complicate direct comparisons [5]. A 2017 review highlighted that PIEB may reduce motor block and improve maternal mobility, but evidence on its impact on delivery outcomes remains inconsistent [6]. Similarly, a 2019 meta-analysis found that PIEB reduced total anesthetic consumption and improved maternal satisfaction compared to CEI, although optimal bolus volumes and intervals require further investigation [7].

Recent studies underscore the need to optimize epidural analgesia to enhance maternal and fetal outcomes. For example, PIEB has been associated with lower rates of instrumental deliveries, potentially due to reduced motor block, but long-term outcomes are rarely explored [8]. Another study suggested that PIEB with ropivacaine and fentanyl may decrease the need for rescue boluses, indicating more consistent analgesia [9]. However, the comparative effectiveness of different anesthetics in CEI versus PIEB remains underexplored, with few studies directly comparing levobupivacaine, bupivacaine, and ropivacaine within standardized protocols [10]. Additionally, the integration of patient-controlled epidural analgesia (PCEA) with CEI or PIEB introduces further variability, as PCEA allows women to self-administer boluses, potentially enhancing autonomy but complicating dosing standardization [11].

Despite advances in epidural techniques, significant gaps persist in the literature. Long-term maternal outcomes, such as postpartum recovery or chronic pain, and neonatal outcomes, including breastfeeding initiation and developmental milestones, are infrequently reported [12]. Furthermore, variations in clinical practice across global obstetric settings and the lack of standardized outcome measures hinder the ability to synthesize findings effectively [13]. This scoping review aims to address these gaps by mapping the current evidence on the efficacy and outcomes of CEI versus PIEB administration of epidural local anesthetics (ropivacaine, levobupivacaine, or bupivacaine) combined with fentanyl or sufentanil for labor analgesia. By synthesizing data on pain relief, motor block, maternal satisfaction, and obstetric outcomes, this review seeks to identify research gaps and provide recommendations for clinical practice and future studies.

## Review

The methodology for the present scoping review adhered to the Preferred Reporting Items for Systematic reviews and Meta-Analyses extension for Scoping Reviews (PRISMA ScR) [14] and the framework of Arksey and O’Malley (2005) [15]. The methodology involved five stages, which are outlined below [16].

Stage 1: Identification of the research question

The purpose of this review was to map the evidence on the efficacy and outcomes of CEI versus PIEB administration of epidural local anesthetics (ropivacaine, levobupivacaine, or bupivacaine) with fentanyl or sufentanil for labor analgesia. The primary research question was, “What is the current evidence on the effectiveness and outcomes of CEI compared to PIEB administration of epidural local anesthetics (ropivacaine, levobupivacaine, or bupivacaine) with fentanyl or sufentanil for labor analgesia?” The secondary objectives were (1) What are the differences in pain relief between CEI and PIEB across these anesthetics? (2) How do CEI and PIEB compare in terms of motor block incidence and severity? (3) What are the impacts of CEI and PIEB on maternal satisfaction and obstetric outcomes? (4) What are the gaps in research regarding optimal dosing regimens, anesthetic comparisons, and long-term outcomes?

Stage 2: Identification of relevant studies

On PubMed, ScienceDirect, and Google Scholar, a comprehensive literature search was conducted between 2015 and March 2025. The search was limited to free full-text studies published in English to capture contemporary evidence relevant to modern epidural analgesia practices. A combination of Medical Subject Headings (MeSH) terms and Boolean operators was used that consisted of (“epidural analgesia” OR “labor analgesia”) AND (“local anesthetic”) AND (“fentanyl”) AND (“continuous infusion” OR “intermittent bolus” OR “programmed intermittent”). The search aimed to identify studies on the efficacy, safety, and outcomes of CEI and PIEB in labor analgesia with these anesthetics and opioids.

Stage 3: Selection of studies

Study selection followed a rigorous process to ensure eligibility. Inclusion criteria were (1) studies comparing CEI and PIEB (or related methods, e.g., PCEA) with ropivacaine, levobupivacaine, or bupivacaine combined with fentanyl or sufentanil; (2) reporting outcomes like pain scores on the Visual Analogue Scale (VAS), motor block (Modified Bromage Score), maternal satisfaction, or obstetric outcomes (e.g., instrumental or cesarean delivery); (3) including healthy nulliparous or parous women at term (≥37 weeks’ gestation); (4) published in English between 2015 and 2025 with full text availability; (5) original research. Excluded were opinion pieces, review articles, editorials, non-peer-reviewed articles, conference abstracts, studies lacking full text or relevance, or those in non-labor settings. The population, concept, and context framework was P = healthy pregnant women at term requesting epidural analgesia, C = efficacy and outcomes of CEI versus PIEB with levobupivacaine, bupivacaine, or ropivacaine and fentanyl or sufentanil, and C = obstetric and anesthesiology settings globally. Two reviewers independently screened titles and abstracts, removing duplicates and irrelevant studies. Full-text articles were assessed, with discrepancies resolved through discussion with a third and fourth reviewer.

Stage 4: Data charting

Data extracted included study characteristics (authors, year, design, location, sample size, and population), intervention details (CEI/PIEB regimens, anesthetic and opioid concentrations, and dosing schedules), findings on pain relief, motor block, maternal satisfaction, obstetric outcomes, research gaps, and recommendations for future research. The methodological quality of the included studies was evaluated using the Mixed Methods Appraisal Tool, a widely used instrument for assessing qualitative, quantitative descriptive (cross-sectional), nonrandomized, randomized controlled trials (RCTs), and mixed methods studies [17]. Studies were classified as high, moderate, or low quality based on the tool’s specified criteria.

Stage 5: Collating, summarizing, and reporting the results

A narrative synthesis was used to summarize findings via text, tables, and figures, with thematic analysis. The results provided an overview of CEI versus PIEB efficacy and outcomes across anesthetics, highlighting differences in pain relief, motor block, maternal satisfaction, and obstetric outcomes. The synthesis identified gaps, particularly in anesthetic comparisons and long-term outcomes, and proposed recommendations for future research and clinical practice.

Results

**Figure 1:**
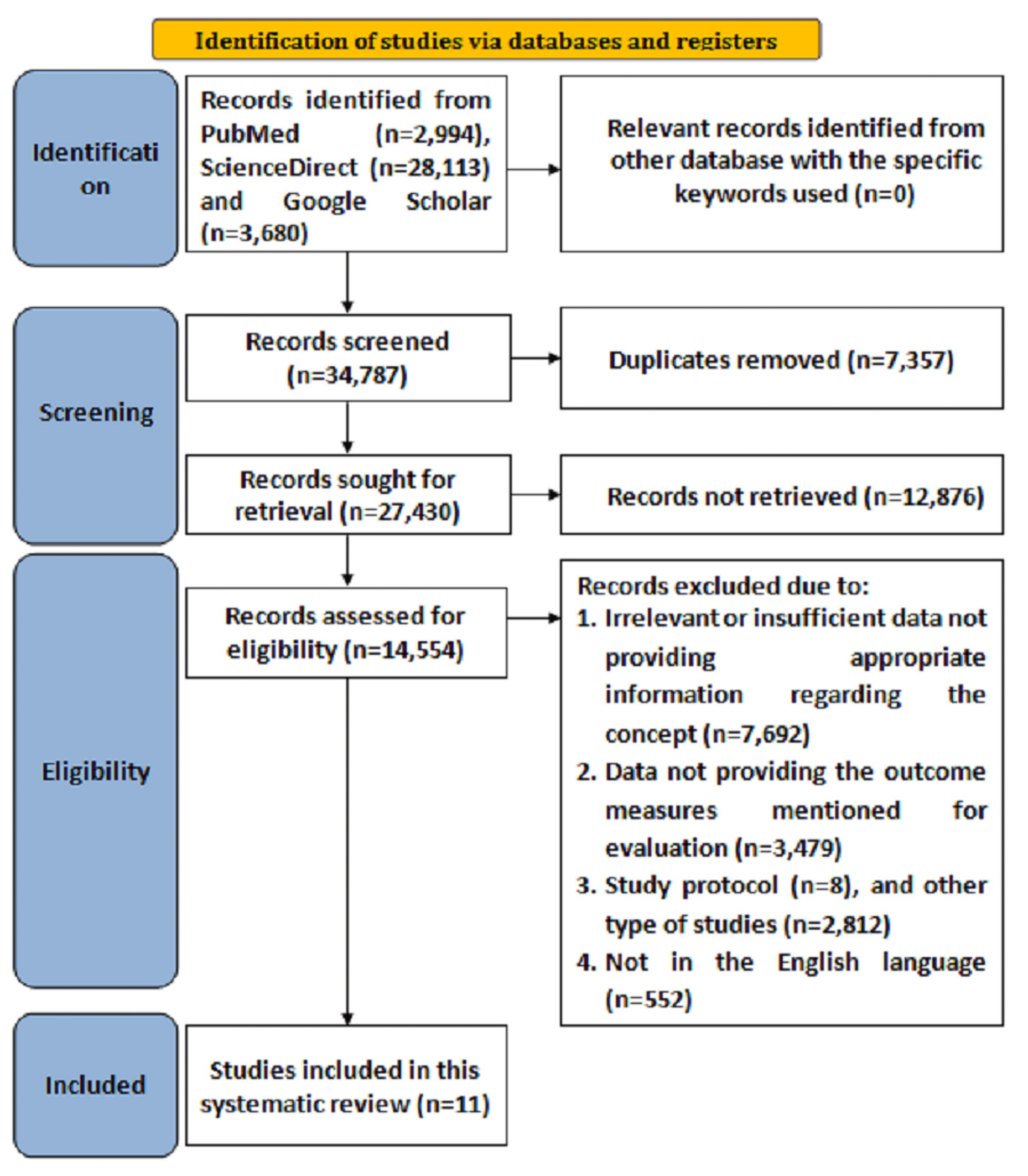
Search strategy

Initially, overall, 34,787 articles were found after searching the databases from 2015 to March 2025 with the specified keywords. Duplicate articles consisting of 7,357 were removed after screening, following which 27,430 articles were sought for retrieval, from which 12,876 articles were not retrieved based on title and abstract evaluation. Moreover, overall, 14,554 records were assessed for eligibility, from which 14,535 records were excluded due to irrelevant data not providing appropriate information regarding the concept (n = 7,692), data not providing the outcome measures mentioned for evaluation (n = 3,479), study protocol (n = 8), other types of studies (n = 2,812), and not being in the English language (n = 552). Hence, for the current review, 11 studies fulfilling the eligibility criteria were included. The Preferred Reporting Items for Systematic reviews and Meta-Analyses (PRISMA) flowchart reporting search strategy is illustrated in Figure 1.

The demographic characteristics and quality of the included studies are reported in Table 1.

**Table 1:** Demographic characteristics of the included studies CEI, continuous epidural infusion; CSE, combined spinal-epidural; IEB, intermittent epidural bolus; NRS, Numerical Rating Scale; PCEA, patient-controlled epidural analgesia; PIEB, programmed intermittent epidural bolus; RCT, randomized controlled trial

Author and year	Study design	Location	Sample size	Study population	Intervention	Quality of the studies
Patkar et al. (2015) [18]	RCT	India	90	Nulliparous, term, aged 20-30 years	CEI group: initial bolus of 10 mL (0.1% ropivacaine + 2 µg/mL fentanyl), followed by continuous infusion at 10 mL/h. IEB group: initial bolus of 10 mL (same composition), followed by intermittent boluses of 5 mL every 30 minutes	High
Tien et al. (2016) [19]	Retrospective	USA	528	Nulliparous and parous women at term	CEI versus PIEB. Both groups: 0.125% bupivacaine with 2 µg/mL fentanyl. CEI group: continuous infusion at 10 mL/h, with PCEA boluses of 5 mL (lockout 10 minutes, max 20 mL/h). PIEB group: programmed boluses of 10 mL every 60 minutes, with PCEA boluses of 5 mL (lockout 10 minutes, max 20 mL/h). Both groups: Initial loading dose of 10-15 mL of 0.125% bupivacaine + fentanyl	High
McKenzie et al. (2016) [20]	Retrospective	USA	400	Nulliparous and parous women at term	CEI versus PIEB. Both groups: 0.125% bupivacaine with 2 µg/mL fentanyl. CEI group: continuous infusion at 12 mL/h, with PCEA boluses of 6 mL (lockout 10 minutes, max 24 mL/h). PIEB group: programmed boluses of 10 mL every 60 minutes, with PCEA boluses of 6 mL (lockout 10 minutes, max 24 mL/h). Both groups: initial loading dose of 15 mL of 0.125% bupivacaine + fentanyl	High
Lin et al. (2016) [21]	RCT	China	200	Nulliparous women at term	PIEB with PCEA versus CEI with PCEA. Test dose (both groups): 1% lidocaine, 4 mL. Initial epidural dose (both groups): 0.15% ropivacaine 10 mL 1. Maintenance (both groups): 0.1% ropivacaine + sufentanil 0.3 µg/mL. IEB group: 5 mL bolus of 0.1% ropivacaine + 0.3 µg/mL sufentanil every 60 minutes, PCEA bolus of 5 mL (lockout 20 minutes), basal infusion rate 0 mL/h, maximum total infusion 15 mL/h. CEI group: continuous infusion at 5 mL/h of 0.1% ropivacaine + 0.3 µg/mL sufentanil, PCEA bolus of 5 mL (lockout 20 minutes)	High
Ferrer et al. (2017) [22]	RCT	Chile	132	Nulliparous and parous women at term	CEI versus PIEB. Both groups: 0.1% bupivacaine with 1 µg/mL fentanyl. CEI group: initial bolus of 12 mL (0.1% bupivacaine + 1 µg/mL fentanyl), followed by continuous infusion at 10 mL/h, with PCEA boluses of 5 mL (lockout 15 minutes). PIEB group: Initial bolus of 12 mL (same composition), followed by programmed boluses of 8 mL every 40 minutes, with PCEA boluses of 5 mL (lockout 15 minutes)	High
Baliuliene et al. (2018) [23]	RCT	Lithuania	237	Nulliparous women at term	PCEA with PIEB for all groups. Four groups: Group 1: 0.08% bupivacaine + 2 µg/mL fentanyl 1. Group 2: 0.1% bupivacaine + 2 µg/mL fentanyl. Group 3: 0.08% levobupivacaine + 2 µg/mL fentanyl. Group 4: 0.1% levobupivacaine + 2 µg/mL fentanyl. All groups: initial bolus of 12 mL (respective anesthetic + 2 µg/mL fentanyl), followed by PIEB of 6 mL every 45 minutes, with PCEA boluses of 5 mL (lockout 10 minutes, max 20 mL/h)	High
Fidkowski et al. (2019) [24]	RCT	USA	150	Nulliparous and parous women	PIEB with PCEA versus CEI with PCEA. Initial epidural dose (both groups): 0.125% bupivacaine 10 mL + fentanyl 100 µg. Maintenance (both groups): 0.0625% bupivacaine + fentanyl 2 µg/mL. PIEB group: 10 mL bolus of 0.0625% bupivacaine + 2 µg/mL fentanyl every 45 minutes starting 30 minutes after initial dose, PCEA bolus of 8 mL (lockout 10 minutes, maximum 32 mL/h including PCEA and PIEB). CEI group: Continuous infusion at 12 mL/h of 0.0625% bupivacaine + 2 µg/mL fentanyl starting 30 minutes after initial dose, PCEA bolus of 8 mL (lockout 10 minutes, maximum 32 mL/h including CEI and PCEA). Breakthrough pain: for pain score ≥ 4 (0-10 scale), 0.125% bupivacaine 5 mL bolus; repeated once if needed; if pain persisted, catheter replaced	High
Ojo et al. (2020) [25]	RCT	USA	120	Nulliparous and parous women at term	CEI versus PIEB. Both groups: 0.1% bupivacaine with 2 µg/mL fentanyl. CEI group: initial bolus of 10 mL (0.1% bupivacaine + 2 µg/mL fentanyl), followed by continuous infusion at 10 mL/h, with PCEA boluses of 6 mL (lockout 10 minutes, max 30 mL/h). PIEB group: initial bolus of 10 mL (same composition), followed by programmed boluses of 10 mL every 60 minutes, with PCEA boluses of 6 mL (lockout 10 minutes, max 30 mL/h)	High
Roofthooft et al. (2020) [26]	RCT	Belgium	130	Nulliparous and parous women at term	PCEA versus PIEB. ropivacaine 0.12% with sufentanil 0.75 µg·mL⁻¹	High
Chalekar et al. (2021) [27]	RCT	India	60	Primiparous women, aged 18-35 years	CEI versus PIEB. Both groups: 0.15% ropivacaine with 2 µg/mL fentanyl. Initial dose (both groups): 12 ml bolus of 0.15% ropivacaine + 50 µg fentanyl (delivered over 10 minutes), with an additional 5 mL of 0.15% ropivacaine + 2 µg/mL fentanyl if T10 sensory level not achieved after 30 minutes. PIEB group: After one hour, 8 mL of 0.15% ropivacaine + 2 µg/mL fentanyl hourly. CEI group: continuous infusion of the same solution immediately after the initial bolus. Breakthrough pain (both groups): 8 mL of 0.15% ropivacaine + 2 µg/mL fentanyl for VAS ≥4	High
Kim et al. (2024) [28]	RCT	South Korea	85	Nulliparous women	CEI with PCEA, PIEB with PCEA, and manual bolus with PCEA. Initial spinal dose (all groups): 0.2% ropivacaine 3 mg + fentanyl 20 µg, CSE. Epidural solution (all groups): 0.2% ropivacaine (60 mL) + fentanyl 180 µg + 0.9% saline (40 mL), yielding approximately 0.12% ropivacaine + 1.8 µg/mL fentanyl. Continuous group: basal CEI at 10 mL/h started 30 minutes after CSE, PCEA bolus of 5 mL (no lockout specified), continued regardless of bolus. PIEB group: PIEB of 10 mL every 60 minutes (240 mL/h flow rate) started 60 minutes after CSE, PCEA bolus of 5 mL (no lockout specified), and PIEB injected 15 minutes after PCEA bolus. Manual group: provider-administered bolus of 10 mL every 60 minutes (1200 mL/h flow rate) started 60 minutes after CSE, PCEA bolus of 5 mL (no lockout specified), manual bolus at set intervals regardless of PCEA. Breakthrough pain (all groups): 0.2% ropivacaine 14 mg (7 mL) epidural bolus for NRS ≥4; if unresolved, 50 mg of 1% lidocaine; cesarean section if labor failed to progress after four hours or per maternal request	High

Moreover, the various findings observed in outcome measures, research gaps, and recommendations for improving future practice are reported in Table 2.

**Table 2:** Summary of the included studies CEI, continuous epidural infusion; IEB, intermittent epidural bolus; NRS, Numerical Rating Scale; PCEA, patient-controlled epidural analgesia; PIEB, programmed intermittent epidural bolus; RCT, randomized controlled trial; VAS, Visual Analogue Scale

Author and year	Findings observed in outcome measures (pain and motor block)	Findings observed in outcome measures (maternal and fetal outcomes)	Research gaps	Recommendations for future research
Patkar et al. (2015) [18]	Both CEI and IEB significantly reduced VAS scores from baseline within 15 minutes post-epidural (p < 0.05). The IEB group had lower VAS scores at two hours and four hours, indicating better sustained pain relief. Moreover, the IEB group had lower motor block incidence: in CEI (p = 0.04), most motor block cases were mild (Bromage score 1). No severe block (Bromage score 3) reported.	The IEB group reported higher satisfaction. Moreover, instrumental delivery rates were lower in the IEB group and were not significant. Cesarean delivery rates were similar. The duration of the second stage of labor was shorter in the IEB group.	Optimal dosing regimens were not reported, and dose-response relationships for ropivacaine were not explored. The study focused on ropivacaine without comparing it to levobupivacaine or bupivacaine, limiting insights into relative efficacy or safety profiles of different anesthetics in IEB versus. CEI. Moreover, no data on long-term maternal or neonatal outcomes (e.g., postpartum recovery, neonatal Apgar scores beyond five minutes, and breastfeeding initiation) were reported. Follow-up was limited to delivery.	Optimize PIEB dosing parameters by conducting RCTs to test varying PIEB bolus volumes and intervals with bupivacaine to identify regimens that minimize motor block while maximizing pain relief, addressing the fixed dosing limitation. Compare anesthetics directly by designing multicenter RCTs comparing bupivacaine, levobupivacaine, and ropivacaine in identical PIEB and CEI protocols to assess differences in VAS, motor block, and obstetric outcomes, filling the gap in anesthetic-specific data.
Tien et al. (2016) [19]	The PIEB group had lower mean VAS scores at two hours and four hours, indicating better pain relief. PIEB required fewer PCEA bolus requests, suggesting more consistent analgesia. The PIEB group had a lower motor block incidence, with most cases mild (Bromage score 1).	Not directly measured as a satisfaction scale. However, indirect proxy via PCEA bolus requests and breakthrough pain indicated higher satisfaction in the PIEB group with fewer breakthrough pain episodes. Instrumental delivery rates were lower in the PIEB group, although not significantly. Cesarean delivery rates were similar in both groups, and the duration of labor was not significantly different.	The study used a fixed PIEB regimen without exploring alternative bolus volumes or intervals, limiting insights into optimal PIEB dosing for bupivacaine. Additionally, it focused solely on bupivacaine, with no comparison to levobupivacaine or ropivacaine, precluding evaluation of anesthetic-specific efficacy or safety in PIEB versus CEI. Moreover, no data on long-term maternal outcomes (e.g., postpartum pain and recovery) or neonatal outcomes (e.g., Apgar scores beyond five minutes and breastfeeding) were reported. Follow-up ended at delivery.	Optimize PIEB dosing parameters by conducting RCTs to test varying PIEB bolus volumes and intervals with bupivacaine to identify regimens that minimize motor block while maximizing pain relief, addressing the fixed dosing limitation. Compare anesthetics directly by designing multicenter RCTs comparing bupivacaine, levobupivacaine, and ropivacaine in identical PIEB and CEI protocols to assess differences in VAS, motor block, and obstetric outcomes, filling the gap in anesthetic-specific data. Moreover, evaluate long-term outcomes by extending follow-up to 6-12 weeks postpartum to study maternal recovery, chronic pain, and neonatal outcomes like breastfeeding initiation and developmental milestones, addressing the lack of long-term data. Enhance study designs by executing prospective RCTs with larger sample sizes (>1,000) to increase statistical power for obstetric outcomes, overcoming the retrospective design’s limitations.
McKenzie et al. (2016) [20]	The PIEB group had lower median VAS scores, indicating better pain control. PIEB required fewer PCEA bolus requests, suggesting more consistent analgesia. The PIEB group had lower motor block incidence, with all cases mild (Bromage score 1).	The PIEB group reported higher satisfaction. Instrumental delivery rates were lower in the PIEB group. Cesarean delivery rates were similar in both groups, and the duration of labor was not significantly different.	Optimal dosing regimens: The study used a fixed PIEB regimen without exploring alternative bolus volumes or intervals, limiting insights into optimal dosing for bupivacaine with fentanyl. The study focused solely on bupivacaine, with no comparison to levobupivacaine or ropivacaine, precluding evaluation of anesthetic-specific efficacy or safety in PIEB versus CEI. Moreover, no data on long-term maternal outcomes (e.g., postpartum recovery, chronic pain) or neonatal outcomes (e.g., Apgar scores beyond five minutes, breastfeeding initiation) were reported. Follow-up ended at 24 hours post-delivery.	Optimize PIEB dosing parameters by conducting RCTs to test PIEB bolus volumes and intervals with bupivacaine and fentanyl to identify regimens that minimize motor block while maximizing pain relief, addressing the fixed dosing used here. Design multicenter RCTs comparing bupivacaine, levobupivacaine, and ropivacaine in identical PIEB and CEI protocols to assess differences in VAS, motor block, satisfaction, and obstetric outcomes, addressing the lack of anesthetic comparisons. Evaluate long-term outcomes to study maternal recovery, chronic pain, mental health, and neonatal outcomes (e.g., breastfeeding, developmental milestones), addressing the absence of long-term data.
Lin et al. (2016) [21]	Baseline VAS was found to be similar. The PIEB group had significantly lower VAS scores at later stages. Time to maximum block height (T10) was found to be similar. Total ropivacaine consumption was lower in the PIEB group. Rescue PCEA boluses were lower in the PIEB group.	The study noted PCEA increased satisfaction, but no quantitative data were provided. Mode of delivery: Similar between groups. Duration of first and second stages: similar in both groups. APGAR scores: similar at one minute and five minutes among both groups.	Optimal dosing regimens: The study used fixed PIEB and CEI regimens without testing alternative bolus volumes, intervals, or infusion rates, limiting insights into optimal dosing for ropivacaine with sufentanil. Additionally, it focused solely on ropivacaine, with no comparison to bupivacaine or levobupivacaine, precluding evaluation of anesthetic-specific efficacy or safety in PIEB versus. CEI. Moreover, no follow-up beyond delivery was performed, omitting data on maternal postpartum recovery, chronic pain, or neonatal outcomes (e.g., breastfeeding and developmental milestones).	Optimize PIEB dosing to optimize analgesia and minimize anesthetic use, addressing the fixed dosing limitation. Design multicenter RCTs comparing ropivacaine, bupivacaine, and levobupivacaine in identical IEB and CEI protocols to assess differences in VAS, motor block, satisfaction, and obstetric outcomes, filling the gap in anesthetic-specific data. Evaluate long-term outcomes. Assess motor block and satisfaction to provide comprehensive outcomes, addressing the absence of these data. Increase sample size (>200 per group) in multicenter RCTs to improve power for detecting differences in delivery modes, building on this single-center study.
Ferrer et al. (2017) [22]	The PIEB group had lower VAS scores at two hours and four hours, indicating better pain relief. PIEB required fewer PCEA boluses, suggesting more effective analgesia. The PIEB group had lower motor block incidence, with all cases mild (Bromage score 1).	The PIEB group reported higher satisfaction. Instrumental delivery rates were lower in the PIEB group, although not significantly. Cesarean delivery rates were found to be similar. The duration of the second stage of labor was found to be shorter in the PIEB group.	Optimal dosing regimens: The study used a fixed PIEB regimen without testing alternative bolus volumes or intervals, limiting insights into optimal dosing for bupivacaine with fentanyl. The study focused solely on bupivacaine, with no comparison to levobupivacaine or ropivacaine, precluding evaluation of anesthetic-specific efficacy or safety in PIEB versus CEI. Moreover, no data on long-term maternal outcomes (e.g., postpartum recovery and chronic pain) or neonatal outcomes (e.g., Apgar scores beyond five minutes and breastfeeding initiation) were recorded. Follow-up was limited to 24 hours post-delivery.	Optimize PIEB dosing parameters to balance pain relief and motor block, addressing the fixed dosing limitation in this study. Compare various anesthetics directly and evaluate long-term outcomes. Increase sample sizes (>200 per group) in multicenter RCTs to improve statistical power for obstetric outcomes. Moreover, explore PCEA integration.
Baliuliene et al. (2018) [23]	No significant differences in VAS scores between groups were observed. All groups achieved effective analgesia. The levobupivacaine 0.08% group had slightly lower VAS at four hours. Lower motor block incidence in groups was observed, and all cases were mild (Bromage score 1).	No significant differences between groups. The levobupivacaine 0.08% group had slightly higher satisfaction. Instrumental delivery rates were similar across groups. Cesarean delivery rates were also found to be similar. Moreover, the second stage of labor duration was similar.	The study used a fixed PIEB regimen without testing alternative bolus volumes or intervals, limiting insights into optimal dosing for bupivacaine or levobupivacaine with fentanyl. Compared bupivacaine and levobupivacaine but only at two concentrations (0.08% and 0.1%) and not against ropivacaine, limiting comprehensive anesthetic-specific efficacy data. No CEI comparison group. No data on long-term maternal outcomes (e.g., postpartum recovery and chronic pain) or neonatal outcomes (e.g., Apgar scores beyond five minutes and breastfeeding initiation) were reported. Follow-up ended at 24 hours post-delivery.	Conduct RCTs to test PIEB bolus volumes and intervals for bupivacaine and levobupivacaine with fentanyl to optimize pain relief and minimize motor block, addressing the fixed dosing used here. Design RCTs comparing bupivacaine, levobupivacaine, and ropivacaine across a wider range of concentrations (e.g., 0.0625-0.125%) in PIEB and CEI protocols to assess differences in VAS, motor block, satisfaction, and obstetric outcomes, building on this study’s limited comparison. Evaluate long-term outcomes, and include a CEI control group. Moreover, investigate PCEA optimization.
Fidkowski et al. (2019) [24]	Baseline pain scores were not reported. No significant difference in pain scores at any time point was provided. Breakthrough pain incidence was found to be similar across groups. Total bupivacaine consumption was lower in the PIEB group. PCEA bolus requests were lower in the PIEB group. No motor block was observed in either group.	No significant difference. The mode of delivery was similar among both groups. Duration of labor was not reported. APGAR scores were not reported.	The study used fixed PIEB and CEI regimens without testing alternative bolus volumes, intervals, or infusion rates, limiting insights into optimal dosing for bupivacaine with fentanyl. Focused solely on bupivacaine, with no comparison to ropivacaine or levobupivacaine, precluding evaluation of anesthetic-specific efficacy or safety in PIEB versus. CEI. No follow-up beyond delivery, omitting data on maternal postpartum recovery, chronic pain, or neonatal outcomes (e.g., breastfeeding and developmental milestones).	Conduct dose-response RCTs to evaluate PIEB bolus volumes and intervals with bupivacaine and fentanyl to optimize analgesia and minimize anesthetic use, addressing the fixed dosing limitation. Design multicenter RCTs comparing bupivacaine, ropivacaine, and levobupivacaine in identical PIEB and CEI protocols to assess differences in VAS, motor block, satisfaction, and obstetric outcomes, filling the gap in anesthetic-specific data. Evaluate long-term outcomes. Assess labor duration and neonatal outcomes, and increase sample size.
Ojo et al. (2020) [25]	The PIEB group had lower VAS scores at two hours and four hours, indicating superior pain relief. PIEB required fewer PCEA boluses, suggesting more effective analgesia. The PIEB group had lower motor block incidence, with all cases mild (Bromage score 1).	The PIEB group reported higher satisfaction. Instrumental delivery rates were lower in the PIEB group. Cesarean delivery rates were similar. The duration of the second stage of labor was shorter in the PIEB group.	The study used a fixed PIEB regimen without testing alternative bolus volumes or intervals, limiting insights into optimal dosing for bupivacaine with fentanyl. Focused solely on bupivacaine, with no comparison to levobupivacaine or ropivacaine, precluding evaluation of anesthetic-specific efficacy or safety in PIEB versus CEI. No data on long-term maternal outcomes (e.g., postpartum recovery, chronic pain) or neonatal outcomes (e.g., Apgar scores beyond five minutes and breastfeeding initiation). Follow-up was limited to 24 hours post-delivery.	Optimize PIEB dosing parameters: compare anesthetics directly, evaluate long-term outcomes, and increase sample sizes to improve statistical power for obstetric outcomes. Investigate PCEA optimization.
Roofthooft et al. (2020) [26]	The PIEB group experienced less frequent breakthrough pain than the PCEA group and had a lower incidence of motor block (modified Bromage score ≤4) compared to the PIEB + PCEA group. The PIEB group used more local anesthetics but required fewer PCEA boluses.	No significant difference.	Fixed PIEB regimens and PCEA settings were not varied, limiting insights into optimal bolus volumes or intervals for levobupivacaine with sufentanil. Used only levobupivacaine, with no comparison to bupivacaine or ropivacaine, restricting evaluation of anesthetic-specific efficacy in PIEB vs. PCEA. No follow-up beyond 24 hours post-delivery, omitting data on maternal postpartum recovery, chronic pain, or neonatal outcomes (e.g., breastfeeding, developmental milestones).	Optimize PIEB and PCEA parameters: Compare various anesthetics, and evaluate long-term outcomes. Increase sample size and explore CEI comparison.
Chalekar et al. (2021) [27]	Both groups achieved complete pain relief (VAS = 0) at 30 minutes and one hour. At two hours, the PIEB group had lower VAS scores. At three hours, no significant difference was observed. No motor block in the PIEB group, and in the CEI group, two parturients had a Bromage score of 4.	The PIEB group had higher satisfaction. Instrumental delivery was more in PIEB. Cesarean delivery was lower in PIEB. Vaginal delivery was more common, and the second stage of labor was significantly shorter in the PIEB group. Positive contraction stress test scores were higher in CEI. Among both groups, APGAR scores at one and five minutes were similar.	The study used a fixed PIEB regimen without exploring alternative bolus volumes or intervals, limiting insights into optimal dosing for ropivacaine with fentanyl. Anesthetic comparisons focused solely on ropivacaine, with no comparison to bupivacaine or levobupivacaine, precluding evaluation of anesthetic-specific efficacy or safety in PIEB versus CEI. No data on long-term maternal outcomes (e.g., postpartum recovery and chronic pain) or neonatal outcomes beyond five-minute APGAR scores (e.g., breastfeeding initiation and developmental milestones) were recorded. Follow-up was limited to 24 hours post-delivery.	Conduct dose-response RCTs to evaluate PIEB bolus volumes and intervals with ropivacaine and fentanyl to balance analgesia, motor block, and obstetric outcomes, addressing the fixed dosing limitation in this study. Compare various anesthetics directly and evaluate long-term outcomes. Increase sample sizes and investigate breakthrough pain management.
Kim et al. (2024) [28]	Baseline and breakthrough pain NRS were similar among groups. However, in the PIEB group, the time to breakthrough pain was longer. Hourly epidural consumption is lower in PIEB. Two cases of motor block were observed: one in continuous (score 4) and one in manual (score 5); none in PIEB (all score 6).	No significant difference. Mode of delivery differed: normal delivery was higher in PIEB; instrumental delivery was zero in PIEB; and cesarean section was less in PIEB. Pairwise comparisons not significant (continuous vs. PIEB; PIEB vs. manual; and manual vs. continuous). Among groups, duration of labor and second-stage duration were also found to be similar. Moreover, among groups, APGAR scores at one and five minutes were also similar..	The study used fixed PIEB and CEI regimens without testing alternative bolus volumes, intervals, or flow rates, limiting insights into optimal dosing for ropivacaine with fentanyl. Focused solely on ropivacaine, with no comparison to bupivacaine or levobupivacaine, precluding evaluation of anesthetic-specific efficacy or safety in PIEB, CEI, or manual bolus methods. No follow-up beyond the immediate post-delivery period, omitting data on maternal postpartum recovery, chronic pain, or neonatal outcomes (e.g., breastfeeding, developmental milestones).	Conduct RCTs to test PIEB bolus volumes, intervals, and flow rates with ropivacaine and fentanyl to balance analgesia, anesthetic consumption, and obstetric outcomes, addressing the fixed dosing limitation. Compare various anesthetics, evaluate long-term outcomes, and increase sample sizes.

Efficacy and Outcomes

Across the included studies, PIEB consistently demonstrated superior pain relief compared to CEI, as measured by lower VAS or Numerical Rating Scale (NRS) scores. This suggests that PIEB provides more consistent analgesia, potentially due to better anesthetic spread in the epidural space, reducing the need for additional boluses. Additionally, when compared to CEI, PIEB was associated with a lower incidence of motor block. This highlights PIEB’s advantage in minimizing motor block, likely contributing to improved maternal mobility during labor. Maternal satisfaction was generally higher in PIEB groups, often linked to better pain control and reduced motor block. This suggests that PIEB enhances maternal satisfaction, potentially due to improved analgesia and mobility, although standardized satisfaction scales are needed for consistency. Moreover, PIEB showed a trend toward improved obstetric outcomes, particularly lower instrumental delivery rates and shorter second-stage labor duration, although results were often statistically nonsignificant. Cesarean delivery rates were generally similar across studies. This indicates that PIEB may reduce instrumental deliveries and shorten labor duration, potentially due to less motor block, but larger studies are needed to confirm statistical significance. In comparison to CEI, PIEB was associated with reduced total anesthetic consumption and fewer rescue boluses. This underscores PIEB’s efficiency in anesthetic use, likely due to optimized drug delivery, which may reduce side effects and costs.

Persistent Research Gaps

The studies consistently identified three major research gaps: (1) Optimal dosing regimens: Most studies used fixed PIEB regimens without exploring variations in bolus volume, interval, or flow rate, limiting insights into optimal dosing [18-28]. (2) Anesthetic comparisons: Most studies focused on a single anesthetic (bupivacaine or ropivacaine), with only Baliuliene et al. (2018) comparing bupivacaine and levobupivacaine, and none including all three (levobupivacaine, bupivacaine, and ropivacaine) in both CEI and PIEB protocols [23]. (3) Long-term outcomes: follow-up was typically limited to 24 hours post-delivery, omitting data on maternal postpartum recovery, chronic pain, or neonatal outcomes like breastfeeding or developmental milestones [18-28]. Additional gaps included small sample sizes. These gaps highlight the need for larger, multicenter RCTs with standardized protocols and extended follow-up.

Discussion

This scoping review of 11 studies [18-28] comparing CEI versus PIEB for labor analgesia, supplemented by broader literature, confirms PIEB’s advantages. PIEB consistently provided superior pain relief, with lower VAS or NRS scores, reduced motor block incidence, enhanced maternal satisfaction, and lowered anesthetic consumption. Trends toward lower instrumental delivery rates and shorter second-stage labor were noted, although often nonsignificant. Hence, these findings align with meta-analyses like Xu et al. (2019) [7], suggesting PIEB’s bolus delivery enhances anesthetic spread, improving sensory blockade and reducing motor effects compared to CEI’s continuous infusion [7].

Patkar et al. (2015) [18] and McKenzie et al. (2016) [20] reported lower VAS scores with PIEB, consistent with Wong et al. (2006), who noted reduced pain scores with PIEB due to higher injection pressure promoting uniform anesthetic distribution [4]. Tien et al. (2016) [19] and Ferrer et al. (2017) [22] found lower motor block rates, aligning with Capogna et al. (2011), which reported a 37% motor block incidence with CEI versus 2.7% with PIEB (p < 0.001) [9]. This likely results from PIEB’s intermittent dosing, minimizing anesthetic accumulation [9]. Baliuliene et al. (2018) [23], focusing on PIEB, suggested lower levobupivacaine concentrations (0.08%) reduced motor block (p = 0.04). Fidkowski et al. (2019) [24] found no VAS or motor block differences, possibly due to low bupivacaine doses (0.0625%), echoing Haidl et al. (2020), where adrenaline-containing solutions diminished PIEB advantages [29]. Maternal satisfaction was higher with PIEB in Ojo et al. (2020) [25], corroborating Xu et al. (2019) (SMD 0.3, p < 0.01), likely due to better analgesia and mobility [7]. Kim et al. (2024) [28] noted longer time to breakthrough pain with PIEB, consistent with a 2019 meta-analysis showing reduced breakthrough pain (OR 0.43, p < 0.05) [7]. Obstetric outcomes, like lower instrumental delivery rates in McKenzie et al. (2016) [20], align with Bullingham et al. (2018), although cesarean rates remained similar [30].

A 2019 meta-analysis found PIEB + PCEA reduced local anesthetic use (-0.74 mg/h ropivacaine equivalents, p < 0.01) and PCEA boluses (OR 0.30, p < 0.01) [7], supporting Lin et al. (2016) [21]. Bullingham et al. (2018) reported less motor block with PIEB (1.0% vs. 21.8%, p < 0.001) and shorter second-stage labor in primiparous women (p < 0.001), suggesting PIEB’s bolus mechanism enhances labor efficiency [30]. Conversely, Haidl et al. (2020) found no significant differences in pain scores or drug consumption with adrenaline-containing solutions (p = 0.08), suggesting solution composition influences outcomes. The mechanism underlying PIEB’s efficacy is higher injection pressure (e.g., 250-500 mL/h), enhancing epidural spread, which is supported by cadaver studies and dye distribution models, contrasting CEI’s slower, less uniform delivery [29].

Limitations

Heterogeneity of study protocols was observed among studies, with varying anesthetic concentrations (e.g., 0.0625-0.2%), bolus volumes (5-10 mL), and intervals (30-60 minutes), complicating direct comparisons. Small sample sizes in some studies limited statistical power, particularly for obstetric outcomes. Most studies focused on short-term outcomes (up to 24 hours post-delivery), omitting long-term maternal or neonatal effects. The review’s reliance on studies from specific regions may limit generalizability to diverse populations. Finally, only one study [23] compared anesthetic types within PIEB, leaving gaps in understanding levobupivacaine, bupivacaine, and ropivacaine differences. Moreover, only free full-text articles were included for the present review, which led to the exclusion of information from other reliable articles.

Recommendations for Future Research

Future research should prioritize large, multicenter RCTs (>200-1000 participants per group) with standardized PIEB protocols (e.g., 8-10 mL boluses every 45-60 minutes) to optimize dosing and confirm obstetric benefits. Comparative studies of bupivacaine, levobupivacaine, and ropivacaine in both CEI and PIEB are needed to clarify efficacy and safety profiles. Extended follow-up (six to 12 weeks postpartum) should assess maternal recovery, chronic pain, and neonatal outcomes. Validated satisfaction scales and consistent pain assessment intervals should be adopted. Investigating PIEB’s cost-effectiveness and workload implications, as raised by some studies, will guide clinical adoption. These efforts will solidify PIEB’s role as a superior labor analgesia method. Hence, these recommendations provide a roadmap for advancing research and clinical practice in epidural analgesia.

## Conclusions

This scoping review synthesizes evidence from 11 studies, demonstrating that PIEB outperforms CEI for labor analgesia across multiple outcomes. PIEB consistently provided superior pain relief, as evidenced by lower VAS/NRS scores, reduced motor block incidence, and enhanced maternal satisfaction, likely due to improved anesthetic spread via bolus delivery. Additionally, PIEB required fewer anesthetics and fewer rescue boluses, suggesting greater efficiency, while trends toward reduced instrumental deliveries and shorter labor duration highlight potential obstetric benefits. However, significant research gaps persist, including limited comparisons of levobupivacaine, bupivacaine, and ropivacaine; fixed dosing regimens; and a lack of long-term maternal and neonatal outcome data. The heterogeneity of study protocols and small sample sizes further limits generalizability. Future research should prioritize large, multicenter RCTs with standardized PIEB protocols to optimize dosing and confirm obstetric advantages. Comparative anesthetic studies and extended follow-up are essential to evaluate efficacy, safety, and long-term impacts, such as maternal recovery and neonatal development. By addressing these gaps, PIEB can be further refined to improve maternal experience and outcomes in labor analgesia globally.
